# Socioeconomic determinants of use of reproductive health services in Ghana

**DOI:** 10.1186/s13561-016-0085-7

**Published:** 2016-02-27

**Authors:** Gordon Abekah-Nkrumah, Patience Aseweh Abor

**Affiliations:** Department of Public Administration and Health Services Management, University of Ghana Business School, P. O. Box 78, Legon, Accra Ghana

**Keywords:** Socioeconomic, Determinants, Reproductive Health

## Abstract

**Background:**

The study examines trends in the consumption of reproductive health services (use of modern contraceptives, health facility deliveries, assisted deliveries, first trimester antenatal visit and 4+ antenatal visits) and their determinants using four rounds of Ghana Demographic and Health Surveys (1993, 1998, 2003 and 2008) data.

**Methods:**

The study uses cross-sectional and pooled probit and negative bionomial regressions models to estimate the determinants of use of the above listed reproductive health services for the period from 1993 to 2008.

**Results:**

Summary statistics suggest that the above-listed reproductive health services have consistently improved from 1993 to 2008. However, use of traditional methods of contraception increased in urban centers between 2003 and 2008, although the reverse was the case in rural areas. Regression results suggest that place of residence, access to and availability of health services, religion, and birth order are significant correlates of use of reproductive health services. Additionally, the study suggests that the number of living children has the largest effect on use of modern contraception. The effect of a partner’s education on use of modern contraception is higher than that of the woman, and a much stronger correlation exists between household wealth and use of reproductive health inputs than expected.

**Conclusion:**

The study associates the increasing use of traditional contraceptives in urban centers and the much stronger effect of household wealth with urban poverty and the increasing indirect cost of health services, and argues for interventions to improve quality of service in public facilities and reduce inequities in the distribution of health facilities. Finally, the study advocates for family planning-related interventions that involve and target partners given the importance of partner education in the use of modern contraception.

## Background

Mother and child health constitute a major challenge in many developing countries. For example, it is estimated that 99 % of all maternal deaths in 2008 were in developing countries [1], with Sub-Saharan Africa (SSA) having the highest maternal mortality rate (MMR) of 640/100,000 live births. In addition, statistics available for under-five mortality and morbidity suggest that developing countries shoulder a higher burden compared to developed countries [2, 3]. Thus, a major objective of primary health care programmes in several developing countries is to improve mother and child survival through improved utilization of preventive reproductive and childcare services [4, 5].

To improve mother and child health, the World Health Organisation (WHO) formulated the Mother Baby Package, based on four principles of safe motherhood: (1) Family Planning – to ensure that individuals and couples have the information and services to plan the timing, number and spacing of pregnancies. (2) Antenatal Care – to prevent complications where possible and ensure that pregnancy-related complications are detected early and treated appropriately. (3) Clean/Safe Delivery – to ensure that all birth attendants have the knowledge, skills and equipment to perform a clean and safe delivery, together with postpartum care for mother and baby. (4) Essential Obstetric Care – to ensure that essential care for high-risk pregnancies and complications is made available to all women who need it.

Following the implementation of the Safe Motherhood programme in many developing countries, and an emphasis on investments in reproductive health inputs as a channel to reducing mother and child-related morbidity and mortality, policy makers and academics have become very interested in the factors that determine the use of reproductive health inputs/services. Thus, over the last two to three decades, substantial research efforts have been directed towards identifying and understanding the factors that influence the use of reproductive health inputs. This notwithstanding, coverage of reproductive health services (especially contraception use and delivery assistance) continues to be low, even when MMR and pregnancy-related malnutrition and complications continue to be high in many SSA countries [1, 6]. For example, the 2008 estimated average MMR for SSA was 640/100,000 live births compared to 85/100,000 for Latin America and the Caribbean (LAC). Although Ghana’s MMR of 350/100,000 live births is deemed to be one of the lowest in SSA, especially when compared to the 1200/100,000 in Chad. Ghana’s figure is nevertheless high compared to 310/100,000 in Bolivia and 17/100,000 in Chile, the highest and lowest respectively in LAC for the same period. The high levels of MMR in the mist low coverage of reproductive health services suggest the need to revisit the use of reproductive health inputs, especially with the availability of more recent datasets.

Although the existing health literature on Ghana abounds in studies that have examined the determinants of reproductive health inputs [7–10], majority of them are either based on a single reproductive health input or on a single cross-sectional dataset. This makes it difficult to see at a glance the changes in the consumption of reproductive health inputs over time and the influence of policy-relevant covariates on several reproductive health inputs. In addition, existing studies have mostly looked at contraception use from an aggregate perspective (i.e., whether a woman uses contraception or not, and whether a woman uses modern contraception or not). We argue that further disaggregation of an input like contraception may be more important in eliciting further information for policy targeting. For example, it is not unreasonable to assume that the effect of socioeconomic factors on the use of contraception will depend on the type of contraception (modern contraception, condoms only, or all other modern contraception other than condoms).

Thus, the current paper pools four rounds of Ghana Demographic and Health Surveys (GDHS) data (i.e., 1993, 1998, 2003 and 2008) and uses that to examine the socioeconomic determinants of use of reproductive health inputs (use of modern contraception, timing of first antenatal visit, number of antenatal visits, health facility delivery and deliveries assisted by health professionals). Specifically, the study first examines changes in the use of the above-mentioned reproductive health inputs across the four surveys. Secondly, the paper examines the socioeconomic determinants of use of the five listed reproductive health inputs through pooled regression estimates. As already indicated, the added value of the current study lies in the fact that the use of four rounds of survey data makes it possible to examine changes in the use of reproductive health inputs across time both at the national, and rural and urban level. Although our regression estimates are based on pooled data, the inclusion of time dummies in the regression model makes it possible to identify a time effect on the use of reproductive health inputs. Thirdly, the disaggregation of use of contraception is important in helping us improve our understanding of the nuanced nature of contraception usage and its determinants.

## Methods

### Data source

The study uses four rounds (1993, 1998, 2003 and 2008) of the GDHS datasets. The Ghana Statistical Service, supported by OR/IFC Macro and IFC International Company, collected all four rounds of the GDHS datasets. The GDHS is nationally representative and based on a two-stage probability sampling strategy. Females aged 15–49 years are interviewed from the selected households. In addition, men aged 15–59 years from a sub-sample of a second or third of total households selected are also interviewed. The survey also collect information on children aged between 0 and 59 months. Information collected by the GDHS survey relevant to the study includes: background characteristics of women and their husbands/partners, reproductive histories, current use of contraceptive methods, antenatal visits, delivery assistance and health facility deliveries. For the purposes of estimating the socioeconomic determinants of use of reproductive health services the different waves (1993, 1998, 2003 and 2008) are pooled. In the case of the descriptive statistics, however, the individual waves are analyzed separately.

### Variable definition and measurement

Modern contraceptives, delivery care and antenatal care are used as indicators of reproductive health services (dependent variables). These three are selected on the basis that they are part of the four services constituting the package of services under the Safe Motherhood programme.

#### Current contraceptive usage

In the survey, women are asked about their current contraceptive use, with the first answer being no use of contraception at all, up to use of about 13 other methods of contraception, that are either modern or traditional. This variable is recoded into three dummy variables (use of modern contraception, use of other modern contraception, that is, all other modern methods excluding condoms and use of only condoms). The three dummy variables are coded 1 where the relevant method is in use, otherwise 0. Traditionally, contraceptive models have been formulated as use of modern or non-modern methods. This is on the basis that non-modern methods are known to be ineffective and therefore could be likened to a situation of not using contraceptives at all. Thus, the decision to disaggregate the variable into the three distinct dummies is to enable us to examine the nuanced nature of the use of the different contraceptive categories (modern, condoms, and other modern methods).

#### Delivery care

Two dummy variables are used to capture delivery care for the last birth preceding the survey. These are deliveries assisted by health professionals (doctors, nurses and midwives) and deliveries occurring in a health facility (private or public). The variables are coded 1 if delivery took place in a health facility or was assisted by any of the three health professionals, otherwise the variable is coded 0. The choice of the two variables is on the basis that they give a woman in labour, access to professional delivery services and emergency obstetric care (EOC) where necessary.

#### Antenatal care

The antenatal visits variable captures the number of antenatal visits made by the pregnant woman (i.e. count form 1,2,3…n). However, WHO recommends at least 4 antenatal visits for a pregnant woman to be deemed protected from pregnancy-related risk and complications [11, 12]. Based on this recommendation, we assume that any number of antenatal visits fewer than 4 is as risky as not going at all. Thus, the variable is coded as binary (1 if a woman had 4+ visits, or else 0). In addition, antenatal visit is used in an ordered and count form to enable us to examine whether the determinants of the intensity of use of antenatal services differ from the determinants of the decision to use or not to use antenatal services. The definition and summary statistics of the remaining variables (i.e., both dependent and independent variables) used are captured in Table 1.

**Table 1:** Summary statistics for use of reproductive health inputs − pooled data: 1993, 1998, 2003, 2008

Variables	Contraceptive models			Variables	Antenatal and delivery		
	N	Mean	SD		N	Mean	SD
Modern contraception	8,270	0.150	0.357	Delivery assistance	8,261	0.476	0.499
Use of condoms only	8,270	0.025	0.155	Health facility	8,259	0.458	0.498
Other modern contracep	8,270	0.125	0.331	Woman’s age			
Woman’s age				15–19 (Ref)	8,261	0.051	0.221
15–19 (1 = base)	8,270	0.051	0.221	20–24	8,261	0.205	0.404
20–24 = (2)	8,270	0.205	0.404	25–29	8,261	0.256	0.436
25–29 = (3)	8,270	0.256	0.436	30–34	8,261	0.208	0.406
30–34 = (4)	8,270	0.208	0.406	35–39	8,261	0.158	0.365
35–39 = (5)	8,270	0.158	0.364	40–44	8,261	0.086	0.281
40–44 = (6)	8,270	0.086	0.281	45–49	8,261	0.036	0.186
45–49 = (7)	8,270	0.036	0.187	Birth order			
Woman’s education				One child (Ref)	8,261	0.208	0.406
No educ (1 = Base)	8,270	0.418	0.493	Two children	8,261	0.195	0.396
Primary = (2)	8,270	0.276	0.447	Three children	8,261	0.158	0.365
Secondary = (3)	8,270	0.293	0.455	Four and above	8,261	0.438	0.496
Tertiary = (4)	8,270	0.013	0.115	Woman’s education			
Partner education				No educ (Ref)	8,261	0.417	0.493
No educ (1 = Base)	8,270	0.328	0.469	Primary	8,261	0.276	0.447
Primary = (2)	8,270	0.168	0.374	Secondary	8,261	0.294	0.455
Secondary = (3)	8,270	0.400	0.490	Tertiary	8,261	0.013	0.115
Tertiary = (4)	8,270	0.063	0.243	Partner education			
Marriage dummy	8,270	0.896	0.306	No educ (Ref)	8,261	0.327	0.469
Muslim dummy	8,270	0.324	0.468	Primary	8,261	0.168	0.374
Ethnicity				Secondary	8,261	0.400	0.490
Akan (1 = Base)	8,270	0.424	0.494	Tertiary	8,261	0.063	0.243
Ga/Dangme = (2)	8,270	0.061	0.239	Missing Husb. Dummy	8,261	0.042	0.200
Ewe and Guans = (3)	8,270	0.145	0.352	Muslim dummy	8,261	0.324	0.468
North ethnicities = (4)	8,270	0.328	0.470	Ethnicity			
Others = (5)	8,270	0.042	0.200	Akan (Ref)	8,261	0.424	0.494
Household wealth				Ga/Dangme	8,261	0.061	0.240
Poorest (1 = Base)	8,270	0.288	0.453	Ewe and Guans	8,261	0.145	0.352
Poorer = (2)	8,270	0.217	0.412	North ethnicities	8,261	0.328	0.469
Middle = (3)	8,270	0.183	0.387	Others	8,261	0.042	0.200
Richer = (4)	8,270	0.171	0.377	Number of elderly	8,261	1.382	0.716
Richest = (5)	8,270	0.141	0.348	Household wealth			
Ecological zones				Poorest (Ref)	8,261	0.288	0.453
Southern belt (1 = Base)	8,270	0.253	0.435	Poorer	8,261	0.217	0.412
Capital city = (2)	8,270	0.092	0.288	Middle	8,261	0.183	0.387
Middle belt = (3)	8,270	0.356	0.479	Richer	8,261	0.171	0.377
Northern belt = (4)	8,270	0.299	0.458	Richest	8,261	0.141	0.348
Rural dummy	8,270	0.708	0.454	Ecological zones			
NSCPHGW	8,270	0.391	0.393	Southern belt (Ref)	8,261	0.253	0.435
NSCPHFT	8,270	0.058	0.165	Capital city	8,261	0.092	0.288
NSCPCCV	8,270	0.717	0.179	Middle belt	8,261	0.357	0.479
No. of living children				Northern belt	8,261	0.299	0.458
No child (1 = Base)	8,270	0.003	0.052	Rural dummy	8,261	0.708	0.455
One child = (2)	8,270	0.233	0.423	NSCPHGW	8,261	0.391	0.393
Two children = (3)	8,270	0.214	0.410	NSCPHFT	8,261	0.058	0.165
Three children = (4)	8,270	0.170	0.376	NSCPCCV	8,261	0.717	0.179
Four and above = (5)	8,270	0.380	0.486	Year			
Year				1993 dummy	8,261	0.219	0.413
1993 dummy (1 = Base)	8,270	0.219	0.413	1998 dummy	8,261	0.256	0.436
1998 dummy = (2)	8,270	0.256	0.436	2003 dummy	8,261	0.291	0.454
2003 dummy = (3)	8,270	0.291	0.454	2008 dummy	8,261	0.235	0.424
2008 dummy = (4)	8,270	0.235	0.424	Sample dummy			
				Timing of 1st antenatal	7514		
				No. antenatal visits	8083		

Source: Authors’ calculations. Calculations take account of sample weights. Note that the models on timing of 1st antenatal visits and number of antenatal visits are based on slightly different samples per the sample dummy. NSCPHGW, NSCPHFT and NSCPCCV are the non-self-cluster proportion of households with good water, non-self-cluster proportion of households with flush toilets, and non-self cluster proportion of children under five with complete vaccination, respectively. The values in parentheses next to the variables are the definitional codes. Note, partner’s education includes a 5th category (missing husbands), which is excluded from the table. This was added to cater for women who do not have partners and would otherwise have been dropped from the regressions

### Statistical estimation

As indicated in Section One, the object of the study is examining the determinants of a woman’s decision to use reproductive health services or not in Ghana. Framing the question in this form reduces the woman’s decision into a binary choice set (i.e. using or not using reproductive health services). If the two alternatives are generalized as *J*, and an indirect utility derived from choosing any of the two alternatives as *V*, then the probability that a woman will use or not use reproductive health services can be expressed as below.

1 $$ \Pr \left({V}_j=1\right)= \Pr \left({X}_j\beta +{\varepsilon}_j>0\right). $$

Where, for instance, (*V*_*j*_ = 1) if reproductive healthcare is used based on the definition of the variables in Table 1, and (*V*_*j*_ = 0) if otherwise. *X* represents a vector of explanatory variables, and *β* are coefficients to be estimated. Consistent with the extant literature, (see for example: [13, 14], *X* is carefully selected to include individual level factors of the women (i.e., age, birth order/number of living children, level of education and that of her partner, marital status, religion and ethnicity), household factors (i.e., household wealth index and number of elderly women in the household) and Community factors (i.e., place of residence and availability and accessibility to health facilities). Unfortunately, the GDHS data does not contains variables (distance to health facility, category of health personnel, and health infrastructure) that have commonly been used as proxies to capture availability and accessibility to health facilities [8, 15, 16].

Thus we follow prior authors [17–19] to compute the non-self cluster proportion of households with access to good water (NSCPHGW), a non-self cluster proportion of households with flush toilets (NSCPHGS), and a non-self-cluster proportion of children with complete vaccinations (NSCPCCV) as proxies for accessibility and availability of health services.

With Equation 1, we are assuming that all dependent variables are binary, including antenatal visits as discussed in Section 2.2. Although the paper’s focus is examining the determinants of use or otherwise of reproductive health services (i.e. binary form), we additionally model the determinants of antenatal care visits in an ordered and count form via an Ordered Probit (OP) and a Negative Bionomial Model (NBM). The use of OP and NBM makes it possible to examine the marginal effect of each additional visit to the threshold of 4+ (in the case of Ordered Probit) or the maximum number of visits (in the NBM).

For the Ordered Probit, antenatal visits are deemed to be in an ordered discrete choice form (1, 2, 3….4+). Thus, the probability that a mother chooses any of the alternatives will increase with utility derived. Assuming there are *I* possible outcomes or antenatal choices facing a mother, a set of threshold coefficients or cut points {*K*_1_, *K*_2_, …, *K*_*I* − 1_} is defined for *K*_0_ = − ∞ and *K*_0_ = ∞, and the choice of antenatal care for the *J*_*th*_ mother may be generalized as:

2 $$ \Pr \left({V}_j=i\right)= \Pr \left({K}_{i-1}<{X}_j\beta +{u}_j<{K}_i\right). $$

Where the probability that individual *j* will choose outcome *i* depends on the attributes of antenatal care and those of the individual/households and community (*X*_*j*_*β*) falling between (*i* − 1). *X* represents a vector of explanatory variables, also defined in Table 1, and *β* are the coefficients to be estimated. The cut-points for the antenatal healthcare choices are based on ordering the number of visits made to the health centre, i.e., ranging from 0 visits, 1 visit… to the maximum number of visits which according to the WHO standards is 4+ for appropriate antenatal care. Thus, Equation 1 is used to estimate all the binary dependent variables, while Equation 2 is used to estimate the determinant of antenatal visits in an ordered form. In the case of the intensity of use of antenatal visits to the maximum number, an NBM is used and the model specification is attached as Appendix 2, with both the estimates of the Ordered Probit and NBM contained in Table 5 in Appendix 1.

## Results

### Descriptive results

**Fig. 1:**
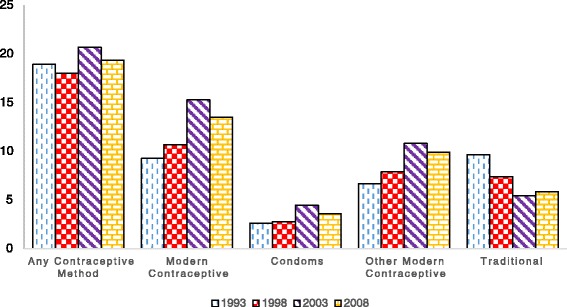
Trends in contraceptive usage - all women (%)

**Fig. 2:**
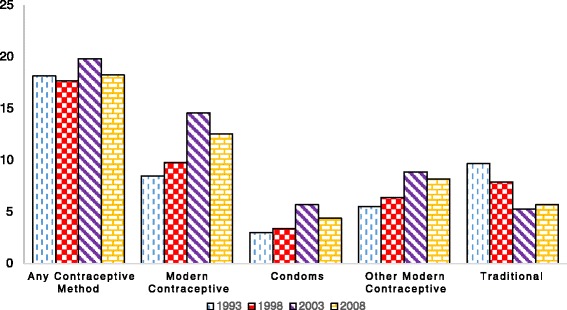
Trends in contraceptive usage - Women under 35 (%)

In this section, we present trends in the use of the three reproductive health inputs at the national and rural/urban areas. Figures 1 and 2 present contraceptive usage at the national level for all women, and women below 34 years of age, respectively. Figures 1 and 2 suggest that the use of modern contraceptives (i.e., any modern method, condoms only and other modern methods) have been improving gradually over the years, except in the case of traditional methods where, as expected, usage is on the decline. Condoms seem to be the least used method of contraception, although the rate of use among women 34 years and below is higher than the average among all women. What is, however, surprising is the fact that apart from traditional methods, use of all other methods of contraception declined between 2003 and 2008. The figures in Table 2 suggest a 19.7 % drop in the use of modern contraceptives between 2003 and 2008. Given that rural consumption continued to increase for the same period, the national level drop in the use of all forms of modern contraception may be attributed to the urban decline in the use of modern contraception.

**Table 2:** Trends in the use of reproductive health inputs in Ghana

Reproductive health inputs		1993	1998	2003	2008
National estimates					
Contraceptive usage	Traditional	90.73	89.34	84.73	86.51
	Modern	9.27	10.66	15.27	13.49
Place of delivery	Home	57.84	54.65	52.06	39.81
	Health facility	42.16	45.35	47.94	60.19
Professional delivery assist	No	56.22	53.78	50.80	36.49
	Yes	43.78	46.22	49.20	63.51
Antenatal visit in 1^st^ trimester	No	61.28	55.89	50.08	42.62
	Yes	38.72	44.11	49.92	57.38
4+ antenatal visits	No	39.95	35.14	28.00	20.00
	Yes	60.05	64.86	72.00	80.00
Urban estimates					
Contraceptive usage	Traditional	87.50	87.27	82.59	86.02
	Modern	12.50	12.73	17.41	13.98
Place of delivery	Home	20.32	22.54	20.69	16.59
	Health facility	79.68	77.46	79.31	83.41
Professional delivery assist	No	18.55	21.91	19.73	14.25
	Yes	81.45	78.09	80.27	85.75
Antenatal visit In 1^st^ trimester	No	55.15	50.88	42.95	37.43
	Yes	44.85	49.12	57.05	62.57
4+ antenatal visits	No	16.88	18.49	10.95	9.73
	Yes	83.12	81.51	89.05	90.27
Rural estimates					
Contraceptive usage	Traditional	92.68	90.50	86.74	86.98
	Modern	7.32	9.50	13.26	13.02
Place of delivery	Home	72.91	65.96	69.52	55.44
	Health facility	27.09	34.04	30.48	44.56
Professional delivery assist	No	71.32	65.01	68.08	51.47
	Yes	28.68	34.99	31.92	48.53
Antenatal visit in 1^st^ trimester	No	64.16	57.80	54.41	46.23
	Yes	35.84	42.20	45.59	53.77
4+ antenatal visits	No	49.14	40.86	37.25	26.88
	Yes	50.86	59.14	62.75	73.12

Source: Authors’ calculation

Note: Calculation takes account of sample weight

In addition to the use of modern contraception, the results in Table 2 suggest that health facility deliveries and deliveries assisted by health professional have been increasing gradually in Ghana. Even when the data is disaggregated into urban and rural areas, health facilities and assisted deliveries continue to show gradual increases, except for the large gap between rural and urban areas. Antenatal care (i.e., antenatal visit in first trimester and 4+ visits) also improved across years, both at the national and disaggregated level (rural/urban). The results in Table 2 equally suggest a marginal rural/urban difference in whether the first antenatal visit occurred in the first trimester, whereas for 4+ antenatal visits, the rural/urban gap remains large.

Although consumption of contraceptives declined for the period 2003 to 2008, the general trend has been that consumption of reproductive health inputs has been better in Ghana compared to many other African countries. For example, Ghana’s percentage of women making 4+ antenatal visits, delivering in a health facility and using modern contraception and condoms in 2008 is relatively better than respective figures in Liberia (66 %, 36.9 %, 11.7 % and 3.5 %), Nigeria (44.8 %, 35 %, 10.5 % and 4.7 %), Sierra Leone (56.1 %, 24.6 %, 8.2 % and 1.1 %), Madagascar (49.3 %, 35.3 %, 23 % and 1 %) and Kenya (47.1 %, 42.6 %, 28 % and 2.6 %). Notwithstanding this, it is also the case that Ghana’s performance compares unfavourably to other developing countries such as Bolivia (72.1 %, 67.5 %, 24 % and 3.6 %), Paraguay (90.5 %, 84.6 %, 52.4 % and 16.3 %) and Jamaica (87 %, 97.6 %, 52 % and 19.4 %) [20].

### Regression results

As earlier indicated, the determinants of use of reproductive health services are estimated using probit models. However, in the case of antenatal visits, additional models; Ordered Probit and Negative Binomial Models (NBM) were used to estimate the marginal effect of every additional visit from 1, 2, 3 and 4+ and 1, 2, 3, 4….n visits respectively. Although the coefficients of the Ordered Probit and NBM were slightly different from that of the probit model, the direction of correlation and level of significance are generally the same. Thus, we present the results of the probit models. In addition, number of living children (NLC) in a contraception consumption model could be endogenous based on reverse causality. A standard correction to this challenge is the implementation of instrumental variable (IV) procedure. However, it is very difficult to find appropriate instruments for endogenous NLC from the DHS data. In the absence of an IV procedure, an alternative is dropping the NLC from the model. However dropping the NLC from the model could potentially result in endogeneity bias arising from omitted variables, especially given the fact that NLC has the highest effect on consumption of contraceptives (see Table 3). For the avoidance of doubt, we have re-estimated the model without NLC. The results (not shown) remain generally the same as including it in terms of direction of correlation and level of significance, but with a drop in the goodness of fit of the model. Thus, we argue that removing the NLC from the contraception model will equally lead to an endogeneity bias, in addition to compromising the goodness of fit of the regression model. Thus, the contraception model uses the NLC as one of the covariates.

**Table 3:** Socioeconomic determinants of contraception use in Ghana

Variables	Sample of all women						Sample of women 34 and below					
	Use of modern contraception		Use of condoms only		Use of other modern methods		Use of modern contraception		Use of condoms only		Use of other modern methods	
	Beta	SE	Beta	SE	Beta	SE	Beta	SE	Beta	SE	Beta	SE
Woman’s age												
20–24	0.0156	[0.0218]	−0.0058	[0.0036]	0.0437*	[0.0262]	0.0161	[0.0212]	−0.0078	[0.0049]	0.0416*	[0.0239]
25–29	0.0035	[0.0219]	−0.0077**	[0.0035]	0.0326	[0.0253]	0.0086	[0.0216]	−0.0108**	[0.0051]	0.0362	[0.0231]
30–34	0.0091	[0.0238]	−0.0070*	[0.0040]	0.0383	[0.0282]	0.0205	[0.0240]	−0.0099*	[0.0056]	0.0493*	[0.0268]
35–39	0.0100	[0.0257]	−0.0129***	[0.0029]	0.0543*	[0.0316]						
40–44	0.0158	[0.0278]	−0.0130***	[0.0024]	0.0643*	[0.0352]						
45–49	−0.0341	[0.0276]	−0.0133***	[0.0020]	0.0107	[0.0357]						
Woman’s education												
Primary	0.0547***	[0.0129]	0.0097**	[0.0046]	0.0429***	[0.0117]	0.0535***	[0.0152]	0.0121*	[0.0064]	0.0398***	[0.0135]
Secondary	0.0654***	[0.0144]	0.0126***	[0.0048]	0.0507***	[0.0133]	0.0739***	[0.0169]	0.0187***	[0.0071]	0.0531***	[0.0152]
Tertiary	0.0723*	[0.0392]	0.0067	[0.0110]	0.0731*	[0.0388]	0.1120**	[0.0539]	0.0138	[0.0186]	0.1114**	[0.0547]
Partner education												
Primary	0.0816***	[0.0183]	0.0096	[0.0070]	0.0732***	[0.0174]	0.0660***	[0.0211]	0.0116	[0.0096]	0.0573***	[0.0196]
Secondary	0.0537***	[0.0140]	0.0130**	[0.0058]	0.0392***	[0.0124]	0.0554***	[0.0171]	0.0217**	[0.0085]	0.0340**	[0.0146]
Tertiary	0.0959***	[0.0259]	0.0379**	[0.0156]	0.0531**	[0.0222]	0.0871***	[0.0311]	0.0550**	[0.0233]	0.0335	[0.0250]
Missing husband dummy	0.0302	[0.0332]	0.0287	[0.0200]	−0.0008	[0.0280]	0.0285	[0.0355]	0.0359	[0.0252]	−0.0048	[0.0281]
Women in union	0.0519***	[0.0129]	0.0087***	[0.0030]	0.0375***	[0.0121]	0.0473***	[0.0154]	0.0093**	[0.0047]	0.0329**	[0.0142]
Muslim dummy	−0.0416***	[0.0100]	−0.0001	[0.0034]	−0.0388***	[0.0091]	−0.0397***	[0.0116]	−0.0017	[0.0046]	−0.0351***	[0.0103]
Ethnicity												
Ga/Dangme	0.0113	[0.0172]	0.0058	[0.0057]	0.0035	[0.0167]	0.0166	[0.0198]	0.0113	[0.0092]	0.0036	[0.0183]
Ewe and Guans	0.0292**	[0.0139]	0.0053	[0.0039]	0.0201	[0.0128]	0.0153	[0.0139]	0.0073	[0.0052]	0.0047	[0.0125]
Northern ethnicities	0.0298*	[0.0161]	−0.0021	[0.0040]	0.0290*	[0.0149]	0.0233	[0.0179]	0.0009	[0.0060]	0.0188	[0.0160]
Others	0.0218	[0.0232]	0.0109	[0.0096]	0.0106	[0.0217]	0.0107	[0.0259]	0.0124	[0.0130]	0.0008	[0.0233]
Household wealth												
Poorer	0.0258**	[0.0127]	0.0129**	[0.0064]	0.0162	[0.0110]	0.0245*	[0.0142]	0.0148*	[0.0085]	0.0130	[0.0120]
Middle	0.0408***	[0.0154]	0.0193**	[0.0080]	0.0253*	[0.0132]	0.0329*	[0.0178]	0.0184*	[0.0096]	0.0183	[0.0151]
Richer	0.0784***	[0.0190]	0.0255**	[0.0102]	0.0538***	[0.0165]	0.0661***	[0.0221]	0.0300**	[0.0130]	0.0383**	[0.0185]
Richest	0.1229***	[0.0264]	0.0354**	[0.0148]	0.0874***	[0.0237]	0.0873***	[0.0278]	0.0423**	[0.0183]	0.0475**	[0.0233]
Ecological zones												
Capital city	0.0000	[0.0169]	0.0103*	[0.0062]	−0.0199	[0.0151]	−0.0325*	[0.0170]	0.0062	[0.0072]	−0.0439***	[0.0147]
Middle belt	0.0167	[0.0113]	−0.0001	[0.0028]	0.0163	[0.0106]	−0.0004	[0.0117]	−0.0024	[0.0037]	0.0023	[0.0108]
Northern belt	0.0180	[0.0179]	0.0017	[0.0050]	0.0141	[0.0162]	0.0146	[0.0201]	0.0039	[0.0074]	0.0089	[0.0177]
Rural dummy	−0.0069	[0.0130]	−0.0042	[0.0033]	−0.0002	[0.0115]	−0.0188	[0.0146]	−0.0062	[0.0045]	−0.0096	[0.0126]
NSCPHGW	−0.0162	[0.0149]	−0.0054	[0.0040]	−0.0081	[0.0139]	−0.0063	[0.0174]	−0.0076	[0.0056]	0.0030	[0.0153]
NSCPHFT	0.0024	[0.0234]	−0.0042	[0.0060]	0.0114	[0.0224]	0.0116	[0.0271]	−0.0050	[0.0083]	0.0200	[0.0250]
NSCPCCV	0.0780***	[0.0266]	0.0045	[0.0064]	0.0700***	[0.0248]	0.0940***	[0.0296]	0.0049	[0.0090]	0.0851***	[0.0273]
No. of living children												
One child	0.9087***	[0.0125]	0.6103***	[0.0479]	0.8706***	[0.0165]	0.8702***	[0.0166]	0.5315***	[0.0479]	0.8131***	[0.0221]
Two children	0.9194***	[0.0112]	0.5551***	[0.0567]	0.9020***	[0.0128]	0.9004***	[0.0144]	0.5112***	[0.0573]	0.8713***	[0.0174]
Three children	0.9320***	[0.0078]	0.6198***	[0.0579]	0.9244***	[0.0095]	0.9298***	[0.0094]	0.6298***	[0.0610]	0.9177***	[0.0125]
Four and above	0.8787***	[0.0152]	0.4386***	[0.0448]	0.8328***	[0.0178]	0.9316***	[0.0101]	0.6870***	[0.0545]	0.9094***	[0.0141]
Year												
1998 dummy	0.0596***	[0.0162]	0.0014	[0.0041]	0.0598***	[0.0156]	0.0368**	[0.0174]	−0.0039	[0.0050]	0.0453***	[0.0166]
2003 dummy	0.1252***	[0.0173]	0.0069	[0.0044]	0.1160***	[0.0173]	0.1129***	[0.0191]	0.0067	[0.0059]	0.1045***	[0.0187]
2008 dummy	0.1035***	[0.0181]	−0.0005	[0.0040]	0.1067***	[0.0179]	0.0801***	[0.0191]	−0.0031	[0.0049]	0.0880***	[0.0187]
*Number of observations*	8270		8270		8270		5955		5955		5955	
Pseudo *R* ^2^	0.074		0.116		0.069		0.074		0.105		0.069	
P-value	0.0000		0.0000		0.0000		0.0000		0.0000		0.0000	

Source: Authors’ calculations

Note: *** is significant at *p* < 0.01, ** is significant at *p* < 0.05, * is significant at *p* < 0.10. NSCPHGW, NSCPHFT and NSCPCCV are the non-self cluster proportion of households with good water, non-self cluster proportion of households with flush toilet and non-self cluster proportion of children under five with complete vaccination, respectively

Partner’s education includes a fifth category (missing husbands) but this is excluded from the table. It was added to cater for women who do not have partners and would otherwise have been excluded from the regressions

It is important to caution that the probit estimates should be interpreted with care given the potential endogeneity of number of living children in the contraception models. It is also important to acknowledge that our quasi R-square is low. However, this in itself is not a challenge given that most relevant variables used in the literature are included in our model and the fact that in general, not much emphasis is often placed on the quasi R-square in a probit model.

As per the results in Table 3 (see estimates for sample of all women), age does not have a significant effect on use of modern contraceptives, although women in the 20–24, 35–39 and 40–44 age brackets are more likely to use other modern contraceptive methods (i.e., modern contraceptive methods other than condoms). Where modern contraceptives is redefined to mean only condoms, all the coefficients on women’s age, with the exception of women in the 20–24 age bracket, become significant with a change in sign from positive to negative. This suggests that compared to younger woman, relatively older women are less likely to use condoms as contraceptives. Even where the model is re-estimated using a sample of women below 34 years of age, the results generally remain the same. Besides contraceptive use, age has a positive correlation with pregnancy-related reproductive health services (i.e., whether the first antenatal visit occurred in the first trimester, 4+ antenatal visits, health facility deliveries and deliveries assisted by health professionals – See Table 4). However, it is important to note that as per the size of the coefficients, the effect of age on consumption of pregnancy-related reproductive health services increases with age, reaches a peak around 40–44 and declines from age 45 and beyond.

**Table 4:** Socioeconomic determinants of antenatal care and delivery care in Ghana

Variables	Antenatal care				Delivery care			
	1^st^ trimester antenatal visit		4+ antenatal visits		Health facility deliveries		Delivery assistance by health professional	
	Beta	SE	Beta	SE	Beta	SE	Beta	SE
Woman’s age								
20–24	0.0684**	[0.0302]	0.0327	[0.0232]	0.0399	[0.0311]	0.0620**	[0.0304]
25–29	0.1529***	[0.0329]	0.1155***	[0.0236]	0.1382***	[0.0343]	0.1630***	[0.0331]
30–34	0.1480***	[0.0342]	0.1303***	[0.0247]	0.1586***	[0.0384]	0.1875***	[0.0374]
35–39	0.1491***	[0.0375]	0.1461***	[0.0238]	0.1769***	[0.0390]	0.2031***	[0.0385]
40–44	0.1634***	[0.0398]	0.1504***	[0.0227]	0.1948***	[0.0411]	0.2044***	[0.0401]
45–49	0.0974**	[0.0430]	0.1250***	[0.0271]	0.1612***	[0.0460]	0.1836***	[0.0455]
Birth order								
2^nd^ birth order	−0.0188	[0.0206]	−0.0637***	[0.0195]	−0.1489***	[0.0207]	−0.1436***	[0.0207]
3^rd^ birth order	−0.0517**	[0.0230]	−0.1230***	[0.0235]	−0.1786***	[0.0232]	−0.1935***	[0.0233]
4^th^ plus birth order	−0.1211***	[0.0241]	−0.1316***	[0.0218]	−0.1956***	[0.0260]	−0.2085***	[0.0270]
Woman’s education								
Primary	0.0280	[0.0182]	0.0625***	[0.0139]	0.0545***	[0.0181]	0.0501***	[0.0180]
Secondary	0.0103	[0.0194]	0.1180***	[0.0168]	0.1461***	[0.0197]	0.1412***	[0.0192]
Tertiary	0.1916***	[0.0601]	0.2217***	[0.0455]	0.2352**	[0.0929]	0.2668***	[0.1021]
Partner education								
Primary	−0.0057	[0.0213]	0.0465***	[0.0171]	0.1100***	[0.0226]	0.1081***	[0.0220]
Secondary	0.0003	[0.0202]	0.0656***	[0.0165]	0.1448***	[0.0200]	0.1401***	[0.0198]
Tertiary	0.0406	[0.0304]	0.1820***	[0.0206]	0.2955***	[0.0324]	0.2871***	[0.0315]
Missing husband dummy	−0.0480	[0.0349]	−0.0690**	[0.0338]	0.0480	[0.0387]	0.0507	[0.0399]
Muslim dummy	−0.0509***	[0.0168]	−0.0881***	[0.0146]	−0.0759***	[0.0189]	−0.0833***	[0.0183]
Ethnicity								
Ga/Dangme	−0.0420	[0.0307]	−0.1263***	[0.0306]	−0.0333	[0.0338]	−0.0374	[0.0330]
Ewe and Guans	0.0174	[0.0176]	−0.0217	[0.0194]	0.0072	[0.0242]	−0.0119	[0.0248]
Northern ethnicities	0.0565**	[0.0244]	0.0401*	[0.0218]	0.0345	[0.0295]	0.0315	[0.0288]
Others	0.1067***	[0.0342]	0.0398	[0.0292]	0.1055***	[0.0357]	0.0872**	[0.0350]
No. of elder women HH	0.0028	[0.0082]	0.0062	[0.0082]	0.0183*	[0.0108]	0.0256**	[0.0112]
Household wealth								
Poorer	0.0370**	[0.0182]	0.0396**	[0.0157]	0.0354*	[0.0199]	0.0341*	[0.0192]
Middle	0.0412**	[0.0201]	0.0494***	[0.0164]	0.1075***	[0.0211]	0.1057***	[0.0205]
Richer	0.0961***	[0.0238]	0.1376***	[0.0178]	0.2099***	[0.0258]	0.2051***	[0.0252]
Richest	0.1806***	[0.0293]	0.1951***	[0.0195]	0.2976***	[0.0308]	0.3051***	[0.0301]
Ecological zones								
Capital city	−0.0426	[0.0282]	0.0647**	[0.0271]	0.1215***	[0.0393]	0.1034***	[0.0377]
Middle belt	−0.0156	[0.0170]	0.0554***	[0.0157]	0.1355***	[0.0199]	0.1354***	[0.0200]
Northern belt	−0.0460*	[0.0265]	0.0995***	[0.0227]	−0.0117	[0.0313]	−0.0109	[0.0311]
Rural dummy	0.0306	[0.0199]	−0.0766***	[0.0188]	−0.2226***	[0.0235]	−0.2289***	[0.0217]
NSCPHGW	0.0319	[0.0255]	0.0492**	[0.0245]	0.0829***	[0.0291]	0.0637**	[0.0286]
NSCPHFT	0.0038	[0.0432]	0.0333	[0.0596]	0.1221*	[0.0664]	0.1601**	[0.0651]
NSCPCCV	0.0290	[0.0401]	0.2363***	[0.0362]	0.1228**	[0.0500]	0.1514***	[0.0488]
Year								
1998 dummy	0.0551**	[0.0240]	−0.0143	[0.0201]	−0.0469*	[0.0263]	−0.0603**	[0.0264]
2003 dummy	0.1040***	[0.0246]	0.0278	[0.0197]	−0.0543*	[0.0290]	−0.0564**	[0.0284]
2008 dummy	0.1917***	[0.0229]	0.0944***	[0.0179]	0.0941***	[0.0291]	0.1231***	[0.0279]
*No. of observations*	7514		8083		8263		8261	
Pseudo *R* ^2^	0.041		0.145		0.277		0.278	
Alpha			985.1906					
Chi2	463.5		0.0000					
P-value	0.0000				0.0000		0.0000	

Source: Authors’ calculations

Note: *** is significant at *p* < 0.01, ** is significant at *p* < 0.05, * is significant at *p* < 0.10. NSCPHGW, NSCPHFT and NSCPCCV are the non-self cluster proportion of households with good water, non-self cluster proportion of households with flush toilet and non-self cluster proportion of children under five with complete vaccination, respectively

Partner’s education includes a 5^th^ category (missing husbands) but this is excluded from the table. It was added to cater for women who do not have partners and would otherwise have been excluded from the regressions

Except for first trimester antenatal visits, women and partners’ education and household wealth are positively and significantly correlated with all the dependent variables for contraception use, antenatal and delivery care. Although both women and partner’s education are significant and positive, the coefficients of partners’ education are slightly higher than that of women’s education in the contraception model. The reverse is true for the antenatal and delivery care models. In addition to the fact that the effect of household wealth is significant and positive, the size of the coefficients increases as one moves from a lower to a higher wealth category. Compared to unmarried women, married women are more likely to use any form of modern contraception, although the probability of use reduces in the case of condoms. In addition, the results also suggest that compared to other religions, Muslim women are less likely to use any form of modern contraception, have their first antenatal visit within the first trimester of pregnancy, have 4+ antenatal visits, deliver in a health facility or to deliver with the assistance of a health professional. Whereas women who have more living children are more likely to use different forms of modern contraceptives, women with 2^nd^ to 4^th^ order births are less likely to use antenatal or delivery care. In the case of birth order, the size of the coefficients increase as a woman moves from a lower order birth to a higher order birth.

The ecological zone and rural dummies are not significant in the contraception models. However, rural women are less likely to have 4+ antenatal visits, deliver in a health facility and have professionally assisted deliveries. In addition, women in the capital city and middle belt are more likely to have 4+ antenatal visits, deliver in a health facility and have professionally assisted deliveries, compared to women in the southern belt. The results also show that women from Northern Ghana are significantly more likely to have 4+ antenatal visits compared to women from Southern Ghana, but less likely to go for antenatal visits in the first trimester (*p* < 0.10), deliver in a health facility or use delivery assistance from health professionals (*p* > 0.10). Also, NSCPCCV is significantly positively correlated with modern contraceptives and other modern contraceptives. In addition, NSCPCCV, NSCPHGW and NSCPHFT are significantly positively correlated with 4+ antenatal visits, health facility delivery and professionally assisted deliveries.

Finally, the coefficients of the year dummies suggest that women were more likely to use modern contraception or any other modern contraception in 1998, 2003 and 2008, respectively, compared to 1993. In the case of antenatal and delivery care, however, the results suggest that women in 1998 and 2003 were less likely to have 4+ antenatal visit, deliver in a health facility and have professionally assisted deliveries compared to women in 1993.

## Discussion of results

The descriptive results suggest that condoms are popular among women 34 years and below compared to all other women. This may be due to the fact that such women are more likely to find alternative contraceptive methods such as pills, injectables, and implants as intrusive and stigmatizing. Additionally, the descriptive results suggest a decline in urban consumption of modern contraceptives. Although reasons for the decline are not directly evident from the data, urban expansion arising from rural–urban migration may provide a plausible explanation. The implications of rural–urban migration may be an increased number of urban dwellers who have characteristics (education and household wealth) similar to rural dwellers. In addition, such migrants often live at the peripheries/fringes of the city or in urban slums where access to health facilities or services are highly constrained. Given such constrained access to health services, lower levels of education and income, it is reasonable to argue that women needing contraceptives may turn to available substitutes such as traditional methods. Indeed, recent evidence from the Multiple Cluster Indicator Survey [21] suggests a decline in urban health facility deliveries at a time when health facility deliveries in rural areas are increasing. Finally the descriptive results show a large rural urban gap in 4+ antenatal visits. This gap may be explained by a variety of factors, including poor road infrastructure, longer average distance to health facilities in rural areas, and the skewed distribution of health facilities and health personnel in favour of urban centres, therefore making it difficult, if not impossible, for women to have access to and consume reproductive health services even when available.

In the case of the regression results, the effect of a woman’s age on use of condoms and other modern contraception is not unexpected. The finding that women above the age of 25 are significantly less likely to use condoms compared to women below 20 may be explained by the fact that younger women who may not have started bearing children are afraid that use of other modern contraceptives (such as injectables, pills and implants) create infertility problems and may therefore not be willing to use them [22, 23]. Conversely, women who are 25 years of age or older are more likely to be married and may need the consent of their partner to use condoms, which are more likely to interfere with sexual relations. Thus, the use of other forms of modern contraception, seen as less interfering sexually, may appeal to such women much more than condoms. In addition, the inverted U-shaped relationship between age and pregnancy-related-reproductive health inputs may be due to the fact that pregnancy complications increases with age, leading to increased consumption of reproductive health inputs among relatively older pregnant women [7, 24]. However, given that reproductive activity reduces at older ages (35–44), it is reasonable to assume that consumption of reproductive health inputs will decline among women of such age group [25, 26].

Women’s education may be a proxy for women’s autonomy; an important determinant of women’s ability to make strategic life choices [27, 28]. These include decisions to use contraceptives, visit the hospital for antenatal care and deliver in a health facility [29]. Similarly, educated women are likely to be more efficient (through access to and use of health-related information) in the production of health compared to their uneducated counterparts [8, 14, 30, 31]. The difference in the size of the women and partners’ education coefficients, although marginal, is still important. As indicated earlier, education may influence household decision-making and, possibly, control of the choice or consumption of reproductive health services. Thus, it may be the case that on matters of contraception, partners have greater control over decision-making [32–35]. Hence, partners who are educated and understand the benefit of contraception use are more likely to exert such influence in the decision to use modern contraception. The higher effect of women’s education on the pregnancy-related reproductive health inputs compared to her partner’s education may be a reflection of access to resources rather than control of decision-making.

The positive effect of household wealth on the use of modern contraception is expected. In Ghana, family planning products are generally controlled by the private sector and are outside the domain of mainstream clinical service providers. Thus, family planning consumables such as condoms, pills and injectable are sold on the market at slightly subsidized prices, making access to resources/wealth an important determinant [35, 36]. In the case of antenatal and delivery care, the positive effect of household wealth is somewhat surprising, especially when one considers the fact that such services are free in public facilities and also covered by the National Health Insurance Scheme. Perhaps the indirect cost of these inputs (distance to health facility and the opportunity cost of visiting a health facility) may be as important as fees paid at the point of service. Alternatively, the poor quality of service at some public facilities may mean that some potential users turn to private providers, who charge market prices and thereby make household wealth an important determinant. The fact that the private health sector in Ghana (Private For Profit Providers, PFPP; Faith-Based Providers, FBP; and Private Non-Profit Providers, PNPP) accounts for around 55 % of health services [37] lends some credence to this suggestion. In addition, an analysis of data from the GLSS 4 (1998/99) and GLSS 5 (2005/06) suggests that the proportion of the respective survey sample who had medical problems and sought help from public facilities dropped from 48 to 45 %, while those who sought help from PFPP and PNPP increased marginally from 47 to 49 %, and 6 to 8 %, respectively [38]. Descriptive results from the GLSS 4 and 5 suggest that 51 and 48 % of the sample, respectively, in the lowest income quintile used services of private providers against 48 and 49 % for those in the richest quintile. Similarly, 48 and 51 % of the sample from rural areas used the services of private providers, against 50 and 47 % of those from urban centres.

In addition, the positive effect of being located in the capital city or the middle belt reflects the resource-rich nature of these zones as well as the concentration of social services such as schools and health facilities, thereby improving access relative to the southern belt. To the contrary, the negative effect of the Northern belt and women living in rural areas reflects a high prevalence of poverty and inadequate infrastructure such as health facilities in rural areas. For example, four rounds of the GLSS – 1991/92, 1998, 2005/06 and 2014 – have consistently cited the Northern belt (Northern, Upper East and Upper West regions) to be the most poverty endermic zone in Ghana. Also, the finding that rural women are less likely to use reproductive health inputs compared to urban women may be due to the fact that in Ghana, as in many developing countries, social infrastructure such as health, water and sanitation facilities tend to be clustered around urban centres. Thus, urban dwellers are more likely to be closer to such facilities and therefore to use them compared to rural women [26, 39, 40].

The fact that married women and women with more living children are more likely to use contraceptives is straightforward and consistent with the existing literature [22, 23]. In addition, the size (largest) of the coefficient of NLC on use of modern contraception is significant: the NLC a woman has is the single most important decision point for the use of contraceptives. This may have undesired implications for population control, especially in a society like Ghana where cultural pressures favour relatively large family sizes. For example, the 2008 GHDS suggests that on the average, a Ghanaian women desires to have four children. For birth order, the negative correlation may be due to the fact that first time/early births are more likely to be associated with pregnancy and birth-related complications. This may explain first timers’ use of more reproductive health inputs compared to women with later order births. It may also be the case that first-timers/women with early order birth may be responding to recommendations from health workers to use reproductive health inputs to reduce the level of risk normally associated with first-time pregnancies [41]. The negative effect on the Muslim dummy, is perhaps an indication that where beliefs associated with the Muslim religion conflict with the demands of modern medicine such as reproductive healthcare, Muslim women may opt not to use it [10, 42]. Not surprisingly, prior authors have found that in Ghana, Muslim woman are less likely to use reproductive health inputs compared to Christian women [7, 9, 10]. The positive effect of the health accessibility and availability proxies (NSCPHGW, NSCPHFT and NSCPCCV) confirms the existing literature [8, 43] that social infrastructure such as health facilities and health personnel are crucial to the consumption of reproductive health inputs.

## Policy implications and conclusion

This study set out to examine the changes in the use of reproductive health inputs (use of modern contraception, and antenatal and delivery care) over time, from 1993 to 2008, as well as the socioeconomic determinants of use of reproductive health inputs. The findings of the study have important implications for reproductive and child health policy formulation. First, the increased use of traditional methods of contraception in urban areas is worrying. It may therefore become important for policy makers to revisit the rural–urban equity narrative in the face of high levels of rural–urban migration, as indicated earlier. The existing narrative that tends to emphasize the fact that rural dwellers are worse off compared to their urban counterparts may lead to resource concentration in rural areas in some cases. For example, the desire in Ghana to bridge the rural–urban gap in the use of reproductive health services, and for that matter reduce MMR in rural areas, led to the adoption and implementation of the Community Health Planning and Services (CHPS) programme in 2003. After about a decade of being implemented, evidence from GDHS 2008 and MICS 2011 suggests that the use of some reproductive health inputs (modern contraception and health facility deliveries) have improved in rural areas at a time when usage is declining in urban centres. Although this paper is not suggesting that the rural–urban consumption difference in the said reproductive health inputs is due to the presence or otherwise of the CHPS programme, it will be equally important for policy makers to relook at how to balance rural–urban resources distribution in a manner that responds to current needs.

Secondly, the fact that the probability of using reproductive health inputs increases with the level of wealth is critical for policy intervention and targeting. As indicated earlier, antenatal and delivery care are generally free and catered for under the National Health Insurance System. Thus, the strong correlation between household wealth and use of antenatal and delivery care suggests that costs other than the direct cost of the services rendered may be very important. Thus, policy makers may need to revisit the discourse on reducing the indirect cost of accessing reproductive health services, which in Ghana is more likely to be associated with the average distance to health facilities and the opportunity cost of visiting the health facilities.

In addition, the fact that the number of living children has the largest effect on the probability of using modern contraceptives in a country where, on the average, women desire to have four children should be an issue for policy attention. The development literature suggest that the desire for large family sizes in developing countries is normally driven by the need for farm hands and sometimes insurance/pensions in old age. Thus, policy measures to modernize agriculture with improved access to subsidized and cheap agricultural technology and inputs, together with appropriate pension schemes especially for rural dwellers, will reduce the desire for large families. Other than the issues of increasing use of traditional contraception in urban areas, the effect of household wealth, partner’s education and number of living children, the effect of the other covariates on the use of reproductive health inputs is standard and supported by the findings of existing studies.

## Appendix 1: Ordered Probit and Negative Binomial Estimates

ᅟ

## Appendix 2: The negative Binomial Model

The negative binomial model improves the efficiency of the Poisson model by adding a parameter, and an error term to the mean function of the Poisson distribution. In the Poisson model, the number of antenatal visits *y*, as in the current case, for a woman *j,* has a Poisson distribution with a mean *μ* conditioned on some covariates *x* as below:

A1 $$ {\mu}_j=E\left({y}_j\left|{x}_j\right.\right)={e}^{x_j\beta },.{y}_j=0,1,2\dots \dots \dots $$

where *β* is the a parameter to be estimated, and the probability of *y* given the vector of covariates *x* expressed as in equation (A2) below :

A2 $$ \Pr \left({y}_j\left|{x}_j\right.\right)=\frac{e^{-{\mu}_j}{\mu}_j^{y_j}}{y_j!},\begin{array}{c}\hfill \hfill \\ {}\hfill .{y}_j=0,1,2,\dots \dots ..\hfill \end{array} $$

In practice, count data often show over-dispersion with the effect that the variance becomes more than the mean and therefore violates the principal Poisson assumption that the variance should be equal to the mean. Apart from the over-dispersion, another challenge of the Poisson model is the issue of excess zeros, which is often associated with count data such as use of antenatal visits. To overcome the two challenges, alternative approaches (such as the Negative Binomial, Zero-Inflated Poisson and Negative Inflated Binomial models) have been used in the literature to account for over-dispersion and excess zeros. In the current case, excess zeros don’t seem to be a challenge given that the percentage of zeros (i.e., those not using) is 12.94 %, 11.20 %, 7.92 % and 3.93 % for 1993, and 1998, 2003 for 2008, respectively. In addition, the variance of antenatal visits is less than the mean in all years. Nonetheless, the lrtest that alpha = 0 (i.e., the Poisson model is an appropriate fit for the data) is rejected at *p* < 0.001 (see column 3 Table 5 in Appendix 1). Thus we use the negative binomial model (NBM) to correct for over-dispersion. The NBM follows from the Poisson model but introduces an additional parameter, an error term *ε*_*j*_, to the mean function of the Poisson distribution as in Equation A3.

A3 $$ {\tilde{\mu}}_j=E\left({y}_j\left|{x}_j\right.\right)={e}^{x_j\beta +{\varepsilon}_j},.{y}_j=0,1,2\dots \dots \dots $$

With the introduction of the error term, the NBM allows for cross-sectional heterogeneity, given that an unobserved individual effect can be taken into the conditional mean function. Following from this, and assuming that exp(e_*j*_) has a distribution with a mean of 1 and a variance of *α* , the conditional mean of *y*_*j*_ will continue to be *μ*_*j*_. Thus as *α* approaches 0, *y*_*j*_ becomes a Poisson distribution. On the other hand, if exp(e_*j*_) is assumed to have a gamma distribution with a gamma function *Γ*(), then the conditional probability function *y*_*j*_ will be the negative binomial distribution as in Equation A4 below.

A4 $$ \Pr \left({y}_j\left|{x}_j\right.\right)=\frac{\varGamma \left({y}_j+{\alpha}^{-1}\right)}{y_j!\varGamma \left({\alpha}^{-1}\right)}{\left(\frac{\alpha^{-1}}{\alpha^{-1}+\mu}\right)}^{\alpha -1}{\left(\frac{\mu }{\alpha^{-1}+\mu}\right)}^{y_j},\begin{array}{c}\hfill \hfill \\ {}\hfill {y}_j=0,1,2\dots \dots \dots \hfill \end{array} $$

Equation A4 was used to estimate the intensity of use of antenatal visits in the count form.

**Table 5:** Socioeconomic determinants of antenatal visits and hospital facility delivery in Ghana

Variables	Ordered antenatal visits		Number of antenatal visits	
	Beta	SE	Beta	SE
Woman’s age				
20–24	−0.0105*	[0.0063]	0.3821**	[0.1742]
25–29	−0.0329***	[0.0062]	1.0020***	[0.1930]
30–34	−0.0351***	[0.0062]	1.1870***	[0.2275]
35–39	−0.0399***	[0.0055]	1.6063***	[0.2515]
40–44	−0.0373***	[0.0051]	1.5979***	[0.2821]
45–49	−0.0336***	[0.0055]	1.5225***	[0.3803]
Birth order				
2^nd^ birth order	0.0244***	[0.0068]	−0.4571***	[0.1006]
3^rd^ birth order	0.0423***	[0.0092]	−0.6750***	[0.1155]
4^th^ plus birth order	0.0437***	[0.0074]	−0.9199***	[0.1264]
Woman’s education				
Primary	−0.0204***	[0.0036]	0.5812***	[0.1126]
Secondary	−0.0360***	[0.0046]	0.8647***	[0.1262]
Tertiary	−0.0499***	[0.0052]	1.0138***	[0.2673]
Partner education				
Primary	−0.0162***	[0.0043]	0.4080***	[0.1506]
Secondary	−0.0201***	[0.0045]	0.5296***	[0.1140]
Tertiary	−0.0455***	[0.0040]	1.1103***	[0.1888]
Missing husband dummy	0.0282**	[0.0121]	−0.2587	[0.2042]
Muslim dummy	0.0332***	[0.0051]	−0.5588***	[0.0977]
Ethnicity				
Ga/Dangme	0.0319***	[0.0104]	−0.6683***	[0.1517]
Ewe and Guans	0.0090	[0.0064]	−0.4239***	[0.1209]
Northern ethnicities	−0.0134**	[0.0061]	0.2082	[0.1521]
Others	−0.0088	[0.0089]	0.1398	[0.2300]
No. of elder women HH	−0.0014	[0.0027]	0.0949	[0.0580]
Household wealth				
Poorer	−0.0118***	[0.0044]	0.2170	[0.1349]
Middle	−0.0166***	[0.0043]	0.5920***	[0.1424]
Richer	−0.0387***	[0.0043]	1.0628***	[0.1635]
Richest	−0.0504***	[0.0047]	1.8244***	[0.2110]
Ecological zones				
Capital city	−0.0154**	[0.0073]	0.5230***	[0.1651]
Middle belt	−0.0178***	[0.0045]	0.3527***	[0.1131]
Northern belt	−0.0210***	[0.0065]	0.2901	[0.1893]
Rural dummy	0.0255***	[0.0052]	−0.5817***	[0.1191]
NSCPHGW	−0.0093	[0.0075]	0.6315***	[0.1586]
NSCPHFT	−0.0149	[0.0184]	0.1153	[0.2191]
NSCPCCV	−0.0788***	[0.0128]	1.6288***	[0.3052]
Year				
1998 dummy	0.0074	[0.0068]	0.1156	[0.1627]
2003 dummy	−0.0116*	[0.0064]	−0.0212	[0.1523]
2008 dummy	−0.0307***	[0.0051]	0.4506***	[0.1550]
* No. of observations*	8083		8083	
Pseudo *R* ^2^	0.098			
Alpha			0.1533	
Chi2	1111.4		1514.0	
P-value	0.0000		0.0000	

Source: Authors’ calculations

Note: *** is significant at *p* < 0.01, ** is significant at *p* < 0.05, * is significant at *p* < 0.10. NSCPHGW, NSCPHFT and NSCPCCV are the non-self cluster proportion of households with good water, non-self cluster proportion of households with flush toilet and non-self cluster proportion of children under five with complete vaccination, respectively. Partner’s education includes a 5^th^ category (missing husbands) but this is excluded from the table. It was added to cater for women who do not have partners and would otherwise have been excluded from the regressions

## References

[1] WHO (2010). Trends in Maternal Mortality: 1990 to 2008 Estimates developed by WHO, UNICEF, UNFPA and The World Bank.

[2] Chopra M, Darnton-Hill I (2006). Responding to the crisis in sub-Saharan Africa: the role of nutrition. Public Health Nutr.

[3] UNDP (2010). Human development report: the real wealth of nations, pathways to human development. Reserch report.

[4] Goldani MZ, Barbieri MA, Silva AAM, Bettiol H (2004). Trends in prenatal care use and low birthweight in Southeast Brazil. Am J Public Health.

[5] Cleland J, Bernstein S, Ezeh A, Faundes A, Glasier A, Innis J (2006). Family planning: the unfinished agenda. The Lancet.

[6] World Bank (2012). World Development Indicators.

[7] Addai I (2000). Determinants of use of maternal and child health services in rural Ghana. J Biosoc Sci.

[8] Overbosch GB, Nsowah-Nuamah NNN, van den Boom GJM, Damnyag L (2004). Determinants of antenatal care use in Ghana. J Afr Econ.

[9] Abor PA, Abekah-Nkrumah G, Sakyi K, Adjasi CKD, Abor J (2011). The socio-economic determinants of maternal health care utilization in Ghana. Int J Soc Econ.

[10] Abekah-Nkrumah G, Guerriero M, Purohit P (2014). ICTs and maternal healthcare utilization. Evidence from Ghana. Int J Soc Econ.

[11] WHO (1994). Antenatal Care: Report of a Technical Working Group.

[12] WHO (1994). Mother baby-pakage: implementing safe motherhood in counries - practical guide.

[13] Glei DA, Goldman N, Rodriguez G (2003). Utilization of care during pregnancy in rural Guatemala: does obstetrical need matter?. Soc Sci Med.

[14] Burgard S (2004). Race and pregnancy-related care in Brazil and South Africa. Soc Sci Med.

[15] Sahn DE (1994). The contribution of income to improved nutrition in Cote d’Ivoire. J Afr Econ.

[16] Lavy V, Strauss J, Thomas D, de Vreyer P (1996). Quality of health care, survival and health outcomes in Ghana. J Health Econ.

[17] Sahn DE, Stifel DC (2002). Parental preferences for nutrition of boys and girls: evidence from Africa. J Dev Stud.

[18] Kabubo-Mariara J, Ndenge GK, Mwabu DK (2009). Determinants of children’s nutritional status in Kenya: evidence from demographic and health surveys. J Afr Econ.

[19] Christiaensen L, Alderman H (2004). Child malnutrition in Ethiopia: can maternal knowledge augment the role of income?. Econ Dev Cult Change.

[20] IFC Macro. Measure DHS STATcompiler. Measure DHS. 2012. http://www.statcompiler.com. Accessed 22 July 2012

[21] Ghana Statistical Service (2011). Ghana multiple indicator cluster survey with an enhanced malaria module and biomarker.

[22] Casterline JB, Sathar ZA, Haque M (2001). Obstacles to contraceptive use in Pakistan: A study in Punjab. Stud Fam Plann.

[23] Hindin MJ, McGough LJ, Adanu RM (2014). Misperceptions, misinformation and myths about modern contraceptive use in Ghana. J Fam Plan Reprod Health Care.

[24] Celik Y, Hotchkiss DR (2000). The socio-economic determinants of maternal health care utilization in Turkey. Soc Sci Med.

[25] Chakraborty N, Islam MA, Chowdhury RI, Bari W, Akhter HH (2003). Determinants of the use of maternal health services in rural Bangladesh. Health Promot Int.

[26] Mekonnen Y, Mekonnen A (2003). Factors influencing the use of maternal healthcare services in Ethiopia. J Health Popul Nutr.

[27] Kabeer N (1999). Resources, agency, achievements: reflections on the measurement of women’s empowerment. Dev Change.

[28] Kabeer N (2005). Gender equality and women’s empowerment: a critical analysis of the third millennium development goal. Gender Dev.

[29] Gabrysch S, Campbell O (2009). Still too far to walk: Literature review of the determinants of delivery service use. BMC Pregnancy Childbirth.

[30] Sahn DE, Younger SD, Genicot G (2003). The demand for health care services in rural Tanzania. Oxford Bull Econ Stat.

[31] Furuta M, Salway S (2006). Women’s position within the household as a determinant of maternal health care use in Nepal. Int Fam Plan Perspect.

[32] Feyisetan BJ (2000). Spousal communication and contraceptive use among the Yoruba of Nigeria. Popul Res Policy Rev.

[33] Moursund A, Kravdal Ø (2003). Individual and community effects of women’s education and autonomy on contraceptive use in India. Popul Stud.

[34] Manlove J, Ryan S, Franzetta K (2007). Contraceptive use patterns across teens’ sexual relationships: The role of relationships, partners, and sexual histories. Demography.

[35] Crissman HP, Adanu RM, Harlow SD (2012). Women’s sexual empowerment and contraceptive use in Ghana. Stud Fam Plann.

[36] Carton TW, Agha S (2012). Changes in contraceptive use and method mix in Pakistan: 1990–91 to 2006–07. Health Policy Plan.

[37] Ministry of Health (2012). Private health sector development policy.

[38] Makinen M, Sealy S, Bitrán RA, Adjei S, Muñoz R (2011). Private health sector assessment in Ghana.

[39] Paredes I, Hidalgo L, Chedraui P, Palma J, Eugenio J (2005). Factors associated with inadequate prenatal care in Ecuadorian women. Int J Gynaecol Obstet.

[40] Allendorf K (2010). The quality of family relationships and use of maternal health-care services in India. Stud Fam Plann.

[41] Navaneetham K, Dharmalingam A (2002). Utilization of maternal health care services in Southern India. Soc Sci Med.

[42] Van Den Heuvel OA, De Mey WG, Buddingh H, Bots ML (1999). Use ofmaternal care in a rural area of Zimbabwe, a population-based study. Acta Obstet Gynecol Scand.

[43] Parkhurst JO, Penn-Kekana L, Blaauw D, Balabanova D, Danishevski K, Rahman SA (2005). Health systems factors influencing maternal health services: a four-country comparison. Health Policy.

